# The Hidden Cause of Maculopapular Rash in Interleukin-12 Deficiency

**DOI:** 10.7759/cureus.21415

**Published:** 2022-01-19

**Authors:** Abdullah M Altamimi, Tala A Qadoumi, Waleed Alajroush, Mohmmed A Alzomia, Ohoud Aljarbou

**Affiliations:** 1 Dermatology, King Abdulaziz Medical City, Ministry of National Guard ‑ Health Affairs, Riyadh, SAU; 2 Dermatology, King Khalid University Hospital, Riyadh, SAU; 3 Pediatric Dermatology, King Abdullah Specialized Children's Hospital, Riyadh, SAU; 4 Internal Medicine, King Abdulaziz Medical City, Ministry of National Guard ‑ Health Affairs, Riyadh, SAU; 5 Pathology and Laboratory Medicine, King Abdulaziz Medical City, Ministry of National Guard ‑ Health Affairs, Riyadh, SAU

**Keywords:** mycobacterium, bcg, salmonella, maculopapular rash, leukocytoclastic vasculitis, interleukin 12

## Abstract

Interleukin-12 RB1 (IL12RB1) deficiency falls under the Mendelian susceptibility to mycobacterial disease. It is a rare genetic disease with autosomal recessive inheritance. It is characterized by recurrent infections with otherwise weak bacteria, such as mycobacteria and *Salmonella*. Often, when encountering a maculopapular eruption, a drug-related cause comes to mind. However, we report a case of IL12RB1 deficiency presenting with a maculopapular eruption, proven by a skin biopsy to be leukocytoclastic vasculitis. The patient was given antibiotics, which improved her skin lesions. Vasculitis should be considered in the differential diagnosis in patients with IL12RB1 deficiency presenting with a cutaneous eruption.

## Introduction

Mendelian susceptibility to mycobacterial disease (MSMD) is a clinical syndrome that puts individuals at risk of infections by mycobacterium species, predominantly the poorly virulent strains such as Bacille Calmette-Guerin (BCG) vaccines and environmental non-tuberculous mycobacteria [1]. They also are susceptible to recurrent salmonellosis, which affects approximately half of the patients, as well as more virulent mycobacterial species [2]. These infections do not normally impact healthy individuals rather patients with certain gene mutations are found to be more susceptible. There are six genes, when mutated, are implicated in the pathogenesis of MSMD. The most common genetic mutation displays autosomal recessive inheritance and affects the gene encoding the β1 chain shared by the IL-12 and IL-23 receptors, resulting in interleukin-12 RB1 (IL12RB1) deficiency [1]. As reported in the literature, patients with MSMD may rarely present with recurrent maculopapular skin lesions [3-4] as a cutaneous manifestation of underlying leukocytoclastic vasculitis (LCV) or Henoch-Schönlein purpura [3]. Furthermore, another cutaneous finding reported in patients with IL12RB1 deficiency is chronic mucocutaneous candidiasis [1]. In this report, we present a case of a patient diagnosed with IL12RB1 deficiency, who developed recurrent maculopapular and purpuric skin lesions, proven by skin biopsy to be LCV.

## Case presentation

**Figure 1:**
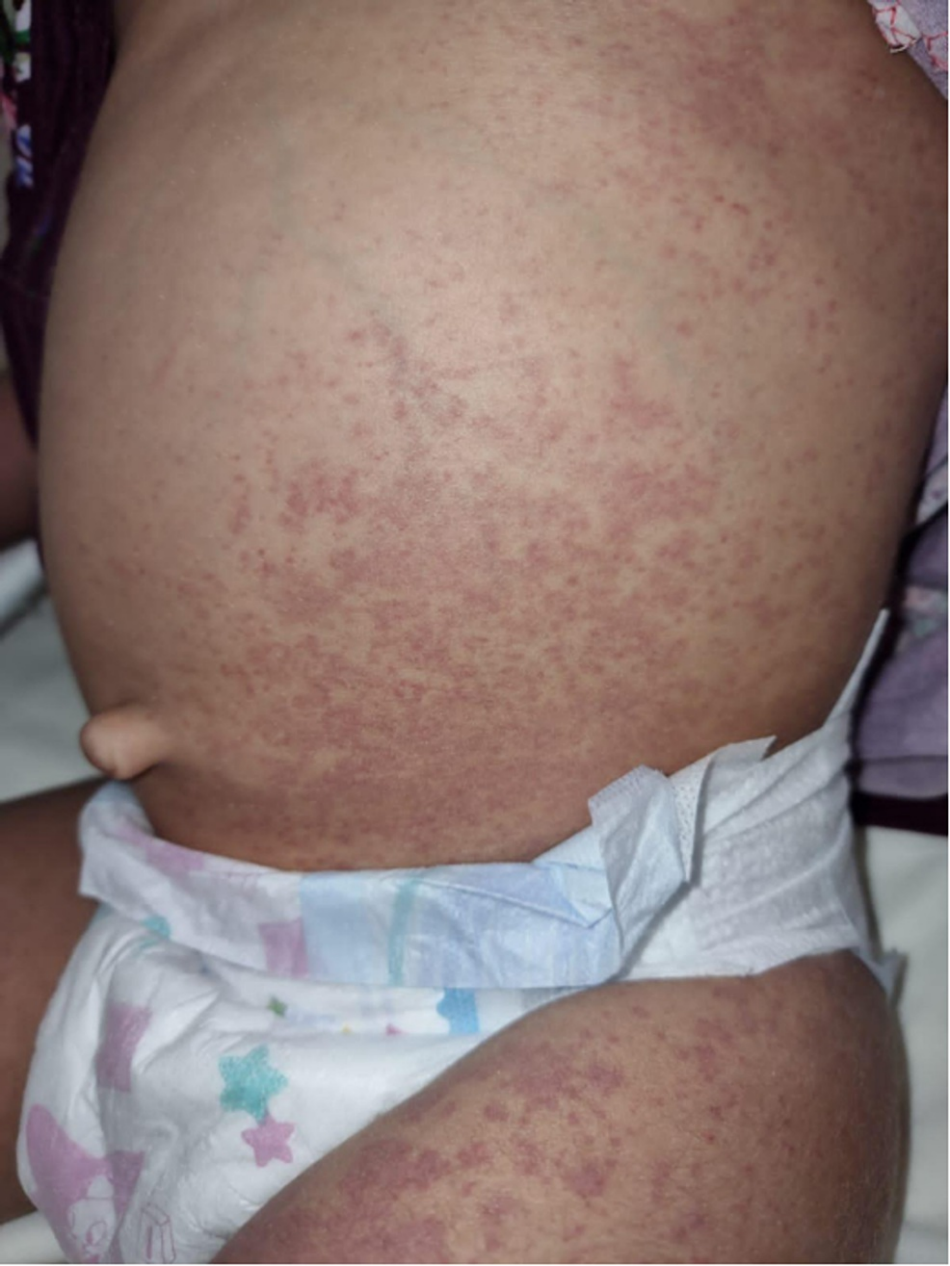
The patient had generalized urticarial macules and papules with few purpuric lesions over the abdomen.

**Figure 2:**
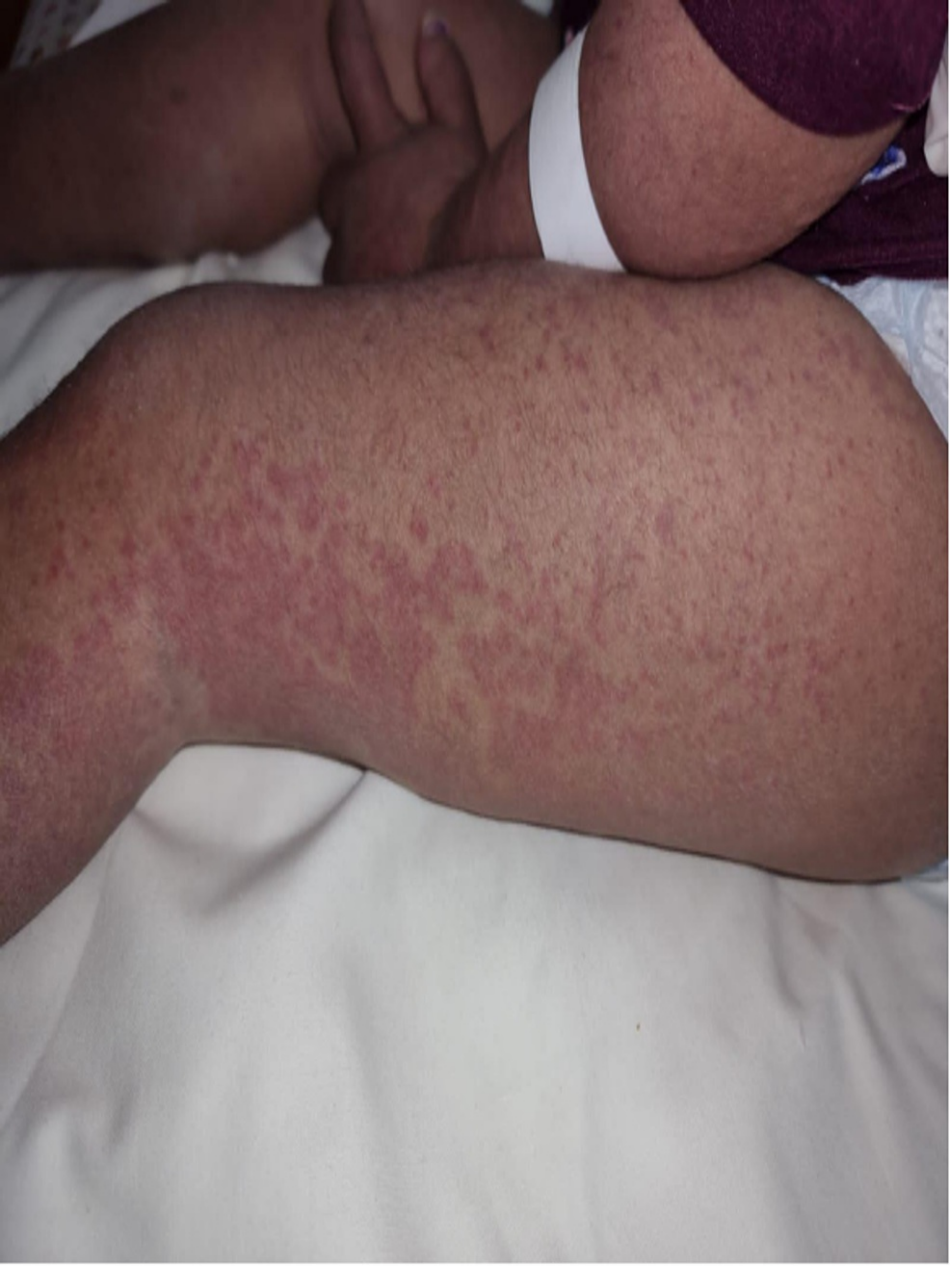
The patient had generalized urticarial macules and papules with few purpuric lesions on the outer thigh.

**Figure 3:**
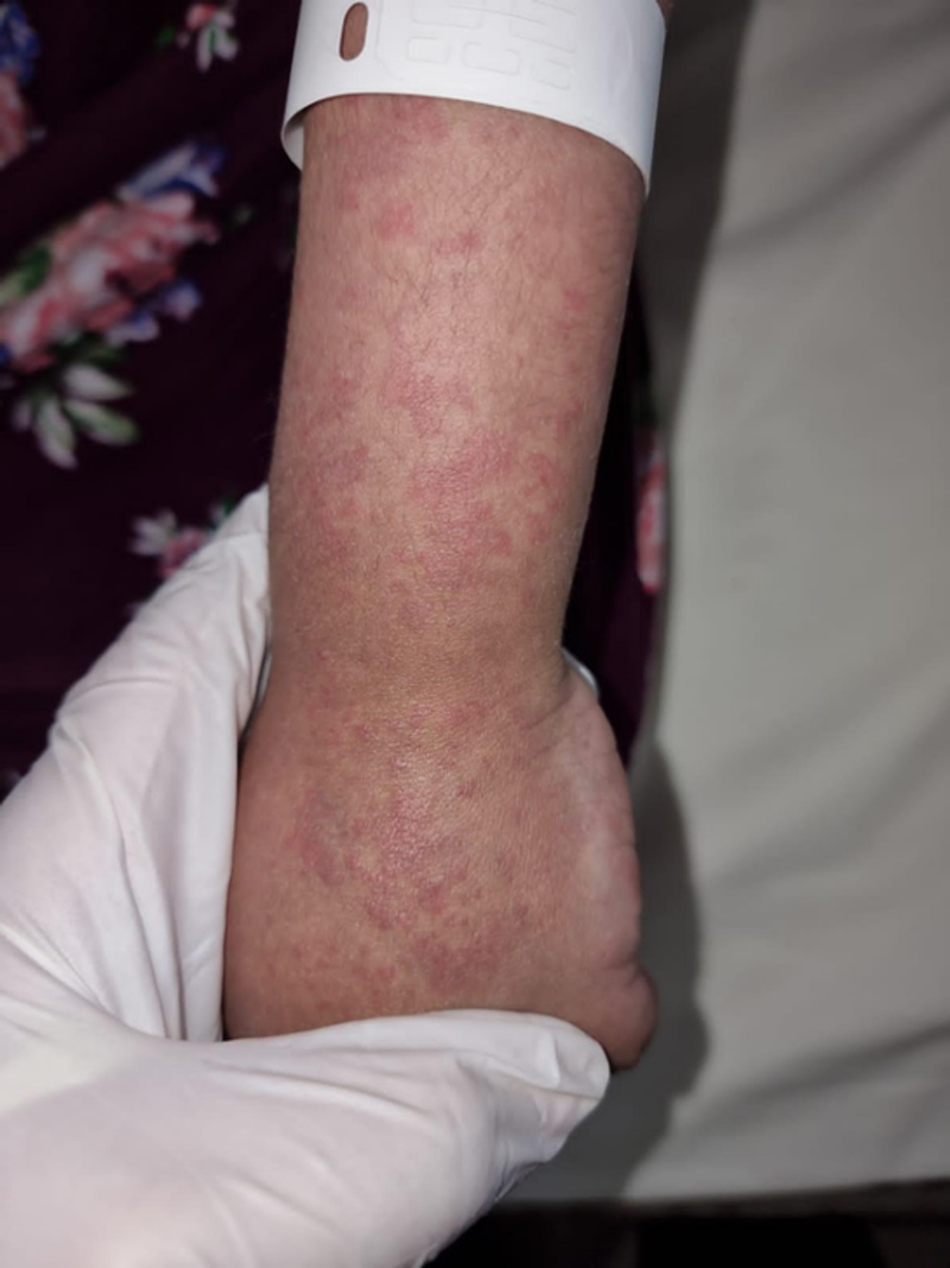
The patient had generalized urticarial macules and papules with few purpuric lesions on the forearm and hand.

**Figure 4:**
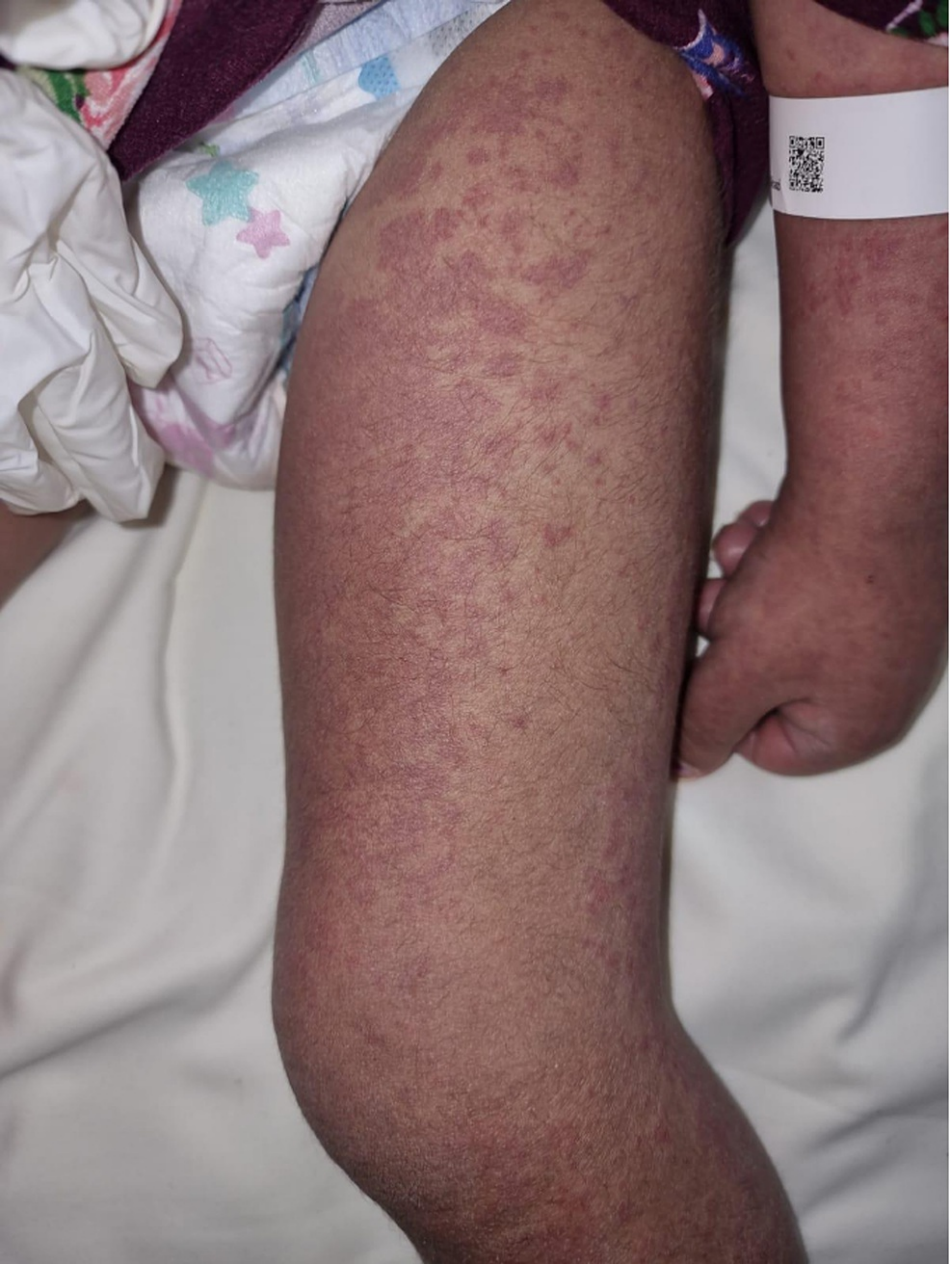
The patient had generalized urticarial macules and papules with few purpuric lesions on the inner thigh.

A four-year-old female presented to King Abdullah Specialist Children’s Hospital Emergency Department with a history of recurrent fevers, productive cough, and a headache for the past two weeks. This patient has a significant past medical history of IL12RB1 deficiency, disseminated BCGitis, and disseminated salmonellosis, with a history of multiple pediatric intensive care unit admissions, secondary to septic shock. She was born to consanguineous parents, and her older sister was also diagnosed with IL12RB1 deficiency. On examination, she was alert, interactive, and not in pain or respiratory distress. The patient had generalized urticarial macules and papules with a few scattered purpuric lesions (Figures 1-4). There were multiple enlarged left cervical lymph nodes and an enlarged left inguinal lymph node, measuring 3x4 cm, with erythema of overlying skin. Chest, cardiovascular, and abdominal examinations were unremarkable. Height and weight growth parameters were normal. Laboratory investigations revealed a hemoglobin level of 59 gm/L. Low blood sodium levels for age-adjusted values were noted, as shown in Table 1. C-reactive protein was 92 mg/L. Coagulation studies, liver panel, creatinine, and blood urea nitrogen all fell within the normal range. COVID-19 swab and blood cultures were taken. Thereafter, the patient was admitted for observation and transfused with 5 mL/kg of packed red blood cells (PRBCs).

**Table 1:** Laboratory findings of complete blood counts, blood chemistry, and coagulation profile of the patient upon presentation. WBC, white blood cells; Hgb, hemoglobin; INR, international normalized ratio; PT, prothrombin time; PTT, partial thromboplastin time; ALT, alanine aminotransferase; AST, aspartate aminotransferase; BUN, blood urea nitrogen; PCR, polymerase chain reaction

Parameter	Result	Normal range
WBC	9.21	4-12x10^9^/L
Hgb	59	110-145 gm/L
Platelet count	180	150-450x10^9^/L
Monocyte count	0.09	0.1-1.1x10^9^/L
Lymphocyte count	1.20	1.40-8.4x10^9^/L
Neutrophil count	7.00	0.8-5.4x10^9^/L
INR	1.08	0.80-1.20
PT	11.90	9.38-12.34 seconds
PTT	26.5	24.84-32.96 seconds
ALT	18	5-55 U/L
AST	32	5-34 U/L
Total bilirubin	5.8	<20.5 umol/L
Alkaline phosphatase	160	156-369
CRP	92	<8 mg/L
Uric acid	352	120-320 umol/L
Total protein	121	60-80 g/L
Potassium	4.8	3.5-5 mmol/L
Albumin	24	38-54 g/L
Sodium	128	138/145 mmol/L
Magnesium	0.92	0.7-0.95 mmol/L
Chloride	104	98-107 mmol/L
Creatinine	42	27-62 umol/L
CO_2_	19	20-28 mmol/L
Random glucose	4.8	3.3-5.6 mmol/L
BUN	4.7	2.5-6 mmol/L
COVID-19 PCR	Negative	

**Figure 5:**
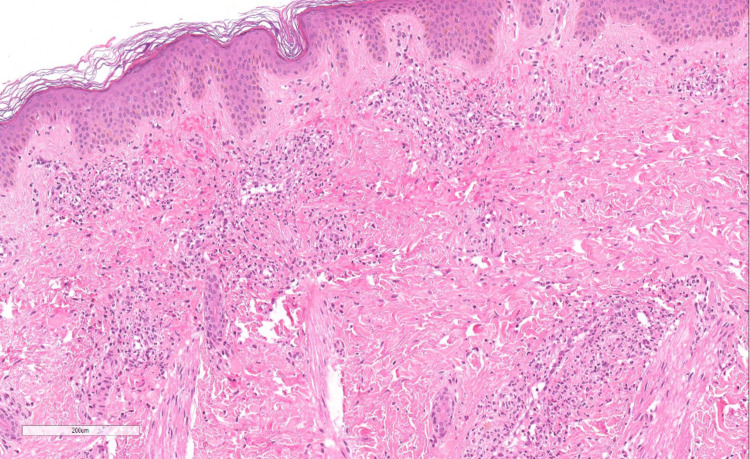
Biopsy revealed perivascular neutrophilic infiltrates involving the superficial and mid vascular plexus associated with nuclear fragmentation and extravasation of red blood cells.

**Figure 6:**
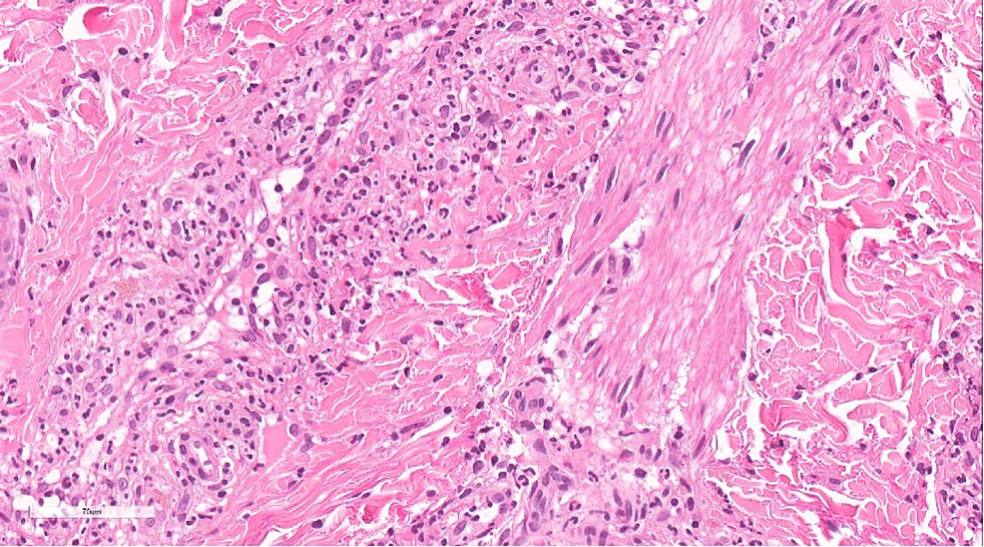
Biopsy revealed perivascular neutrophilic infiltrates involving the superficial and mid vascular plexus associated with nuclear fragmentation and extravasation of red blood cells.

Prior to her admission, she was on cefixime, linezolid, rifampicin, moxifloxacin, multivitamins, and pegylated interferon alfa-2a. The medications were initially continued upon admission, with the exception of cefixime, which was replaced with ceftriaxone. The following day, her hemoglobin was found to be 63 gm/L, and another 5 mL/kg of PRBCs were given. On the third day post-admission, blood cultures grew *Salmonella*. Ceftriaxone was then replaced with meropenem, and the patient's overall condition improved. However, the skin lesions persisted. Previous hospital records showed that the patient developed similar skin lesions with the recurrence of bacteremia, resolving usually within two days, without post-inflammatory hyperpigmentation. The patient's primary team consulted us to further evaluate her skin lesions, as they were under the impression that this could be a drug eruption. A 4-millimeter punch biopsy was taken. The biopsy revealed perivascular neutrophilic infiltrates involving the superficial and mid vascular plexus associated with nuclear fragmentation and extravasation of red blood cells (Figures 5, 6). Diagnosis of LCV was made based on the histopathologic findings.

Blood cultures were repeated five days post-admission and were found to be negative. The patient's skin lesions improved, and she was discharged on linezolid, rifampicin, moxifloxacin, and fosfomycin.

## Discussion

We described a case of a four-year-old female child with IL12RB1 deficiency, presenting with recurrent maculopapular/purpuric skin lesions, proven by skin biopsy to be LCV. These lesions were attributed to the underlying *Salmonella* infection. Most cases reported about IL12RB1 deficiency patients presenting with LCV were thought to be due to an infection with *Salmonella* [5], with the exception of two cases in which there was no clear *Salmonella* infection [3]. In one of the aforementioned exceptions, the authors still presumed the possibility of a subclinical *Salmonella* infection [3]. In this patient, following the fifth day of meropenem, the cutaneous lesions improved, supporting *Salmonella* as the cause of her cutaneous manifestations. Moreover, two case reports published on patients with IL12RB1 deficiency reported similar cutaneous findings. One reported the presence of hemorrhagic-vasculitic lesions on both legs upon admission of the patient [3]. The second case report found a relapsing maculopapular eruption [4]. Both patients also had lymphadenopathy on examination. Another skin manifestation reported in patients with IL12RB1 deficiency is mucocutaneous candidiasis [1].

Disseminated BCGitis is usually the first manifestation of IL12RB1 deficiency after inoculation with the BCG vaccine. The impaired IL-12/IL-23 signaling pathway put such individuals at risk of infections by intracellular organisms, such as mycobacterial and *Salmonella* species. IL-12 is an important signaling molecule in T-helper 1 (Th-1) response and cell-mediated immunity. The loss of expression of IL-12 leads to defective signaling and impaired interferon gamma (IFN-γ) production, which results in a weakened cell-mediated immune response [6]. As we reported in our patient, recurrent infections with salmonellosis and BCGitis are typical in patients with IL12RB1 deficiency, usually with an onset in childhood. Infections with coccidioidomycosis and leishmaniasis have also been reported in such patients, possibly highlighting the role of IL-12 in eliminating these organisms [7-9].

## Conclusions

In conclusion, patients with MSMD, particularly those with IL12RB1 deficiency, are predisposed to infections by opportunistic organisms, namely BCG and *Salmonella*. *Salmonella* may result in LCV with cutaneous manifestations, which improves upon administration of antibiotics. Therefore, LCV may be a presenting feature in patients with IL12RB1 deficiency. Physicians should have a heightened sense of suspicion for IL12RB1 deficiency in children with similar skin lesions and recurrent infections with the classic organisms.
